# Abscisic Acid and Gibberellins Antagonistically Mediate Plant Development and Abiotic Stress Responses

**DOI:** 10.3389/fpls.2018.00416

**Published:** 2018-03-27

**Authors:** Kai Shu, Wenguan Zhou, Feng Chen, Xiaofeng Luo, Wenyu Yang

**Affiliations:** Key Laboratory of Crop Ecophysiology and Farming System in Southwest China, Institute of Ecological Agriculture, Sichuan Agricultural University, Chengdu, China

**Keywords:** seed dormancy, germination, ABA, GA, antagonism, abiotic stress

## Abstract

Phytohormones regulate numerous important biological processes in plant development and biotic/abiotic stress response cascades. More than 50 and 100 years have passed since the initial discoveries of the phytohormones abscisic acid (ABA) and gibberellins (GA), respectively. Over the past several decades, numerous elegant studies have demonstrated that ABA and GA antagonistically regulate many plant developmental processes, including seed maturation, seed dormancy and germination, root initiation, hypocotyl and stem elongation, and floral transition. Furthermore, as a well-established stress hormone, ABA plays a key role in plant responses to abiotic stresses, such as drought, flooding, salinity and low temperature. Interestingly, recent evidence revealed that GA are also involved in plant response to adverse environmental conditions. Consequently, the complex crosstalk networks between ABA and GA, mediated by diverse key regulators, have been extensively investigated and documented. In this updated mini-review, we summarize the most recent advances in our understanding of the antagonistically regulatory roles of ABA and GA in different stages of plant development and in various plant–environment interactions, focusing on the crosstalk between ABA and GA at the levels of phytohormone metabolism and signal transduction.

## Introduction

Various plant hormones play key and distinct roles in the plant life cycle, from seed maturation, seed germination to the floral transition and abiotic/biotic stress responses (Shu et al., 2016c; Yang and Li, 2017). Which phytohormones regulate plant developmental processes and stress adaptation, and what are the mechanisms? In the past decades, tremendous progress has been achieved in the field of plant hormone biology to answer these questions, in particular in the model plant *Arabidopsis thaliana*. The anabolism, catabolism, transport and signal transduction pathways of the phytohormones have been documented (Novak et al., 2017). Numerous elegant studies have demonstrated that different phytohormones interact antagonistically and/or synergistically with one other, forming complicated crosstalk networks (Shan et al., 2012). As a result of such crosstalk networks, different phytohormones regulate distinct biological processes precisely throughout a plant’s life cycle.

Abscisic acid (ABA) and gibberellins (GA) are one pair of classic phytohormones, which antagonistically mediate several plant developmental processes, including seed maturation, seed dormancy and germination, primary root growth, and flowering time control (Wang et al., 2013; Luo et al., 2014; Yang et al., 2014; Shu et al., 2016a,c). Thus, the crosstalk between ABA and GA is a research hotspot in the fields of plant molecular biology and plant genetics, with numerous key regulators, including DELLAs and AP2-domain-containing transcription factors, having been extensively investigated and reviewed (Liu X. et al., 2016; Shu et al., 2018b). These factors carry out functions, central to ABA and GA antagonism, affecting phytohormone biosynthesis and/or signal transduction pathways (Lin et al., 2015; Liu X. et al., 2016; Shu et al., 2018b).

Furthermore, as a near-universal abiotic stress hormone, ABA is involved in diverse abiotic stress response cascades, including those related to drought, flooding, salinity and low temperatures (Zhu et al., 2017). Although GA are primarily regarded as plant growth regulators involved in a number of developmental processes, including stem elongation (Jan et al., 2006; Iwamoto et al., 2011; Li et al., 2011), and flowering time control (Ding et al., 2013; Hyun et al., 2016), recently published data demonstrate that GA also control certain biological processes in response to stress (Qin et al., 2011; Hamayun et al., 2017; Urano et al., 2017; Wang et al., 2017). Consequently, the detailed mechanisms by which ABA and GA precisely mediate plant development and stress responses are ripe for further exploration.

Given the significant progress which has taken place in research into ABA and GA antagonism field, this mini-review will highlight the most recent advances (primarily, over the past 4 years) in the regulatory roles of ABA and GA in plant development and stress responses, focusing on the crosstalk between ABA and GA, as mediated by the key regulators. Finally, future research directions and challenges in this field will be discussed.

## ABA and GA: Focusing on Seed Dormancy and Germination

Seed science is one of the most important research fields in plant molecular biology, including seed maturation, seed dormancy and germination, and seed longevity (Waterworth et al., 2015; Sano et al., 2016; Shu et al., 2016c; Nee et al., 2017). Crop seed is the direct product of the agricultural system, and thus optimal germination and seedling emergence in the field are important to achieve the final yield. ABA and GA play different key roles in the regulation of seed dormancy and germination, and the metabolism of and signaling by both phytohormones also changes during seed development, namely seed maturation, seed dormancy and germination, and seedling establishment (Shu et al., 2015, 2016c).

ABI4 (Abscisic acid-insensitive 4), an enhancer of the ABA signal transduction pathway, deepened seed dormancy in *Arabidopsis* by increasing ABA biosynthesis while decreasing GA biosynthesis (Shu et al., 2013). Detailed biochemical analysis showed that ABI4 directly interacts with promoter regions of *NCED6*, an ABA biosynthesis gene, and of *GA2ox7*, a GA-inactivator gene (Shu et al., 2016b). Consequently, ABI4 is the central factor which mediates the antagonism between ABA and GA by regulating the biosynthesis of both phytohormones, resulting in the precise control of the degree of seed dormancy and post-germination seedling growth (Shu et al., 2016c, 2018b). Interestingly, ABI4 also inhibits seed germination and cotyledon greening through the mediation of cytokinin signaling (Huang et al., 2017). Investigations revealed that, in *Sorghum bicolor*, transcription factors SbABI4 and SbABI5 enhanced the transcription of *SbGA2ox3*, a GA-inactivator gene, through directly binding to its promoter, and consequently extended seed dormancy (Cantoro et al., 2013). During the post-germination seedling growth stage, ABI4 also enhanced *ANAC060* transcription by directly interacting with its promoter, with ANAC060 reducing ABA sensitivity and glucose-mediated ABA accumulation (Li et al., 2014). Another gene, *CK2* (*Casein Kinase 2*), positively mediated ABA signaling and stress responses during seed germination and early seedling establishment, the partial mechanism being that CK2 indirectly enhanced *ABI4* expression (Wang et al., 2014). Furthermore, diverse factors, including miRNA 165/166, E3 ubiquitin ligase CER9 (ECERIFERUM 9), transcription factors RAV1, OsAP2-39 and MYB96, nuclear C2H2 zinc-finger protein ZFP3, and AtGLR3.5 (glutamate receptor homolog 3.5), regulated ABA signaling during seed germination and post-germination seedling growth through the ABI4-mediated cascades (Yaish et al., 2010; Feng et al., 2014; Joseph et al., 2014; Zhao et al., 2014; Kong et al., 2015; Lee et al., 2015; Yan et al., 2016). Consequently, ABI4 is a key factor with regard to ABA-mediated regulation of seed germination and early seedling establishment.

Recently, several key components, which regulate seed germination, were dissected by analyzing their effect on the balance between ABA and GA. GIM 2 (Germination Insensitive to ABA mutant 2) promoted GA biosynthesis while reducing ABA biosynthesis, and subsequently the *gim2* mutant seeds showed the ABA-insensitive phenotype during seed germination and post-germination seedling growth (Xiong et al., 2017). In addition, exogenous auxin or NaCl treatment delayed soybean seed germination through decreasing the GA/ABA ratio (Shu et al., 2017; Shuai et al., 2017). A similar effect of NaCl on seed germination of the halophyte *Suaeda salsa* has been reported, which is also mediated through ABA and GA pathways (Li et al., 2015). Aluminum (Al) in contaminated soil inhibited rice seed germination, while exogenous H_2_ (hydrogen) alleviated the Al toxicity through increasing the GA/ABA ratio (Xu et al., 2017). Further detailed analysis showed that H_2_ promoted the expression of *GA20ox1* and *GA20ox2*, two GA biosynthesis genes, and of *ABA8ox1* and *ABA8ox2*, two ABA catabolism genes (Xu et al., 2017).

In maize, under chilling stress, the combination of the phytohormone salicylic acid (SA) and the reactive oxygen species hydrogen peroxide (H_2_O_2_) up-regulated transcription of both the GA biosynthesis gene *ZmGA20ox1* and the ABA catabolism gene *ZmCYP707A2*, while down-regulating the expression of the GA catabolism gene *ZmGA2ox1* (Li Z. et al., 2017). Exogenous application of SA and H_2_O_2_ increased the GA/ABA ratio and accelerated maize seed germination under chilling stress conditions (Li Z. et al., 2017). Karrikins, a group of plant growth regulators present in the smoke of burning plant material, mediated soybean seed germination through regulating GA and ABA biosynthesis and signal balance (Meng et al., 2016a,b). Moreover, the NF-YC-RGL2-ABI5 cascades, which integrate GA and ABA signaling pathways to precisely regulate seed germination (Liu X. et al., 2016), have been dissected. FOA2 (F-box overexpressed/oppressed ABA signaling), and the transcription factors FUSCA3 and DAG1 (DOF AFFECTING GERMINATION 1) also regulate biosynthesis and signal transduction of GA and ABA during seed germination (Boccaccini et al., 2014a,b, 2016; Chiu et al., 2016a,b; He et al., 2016). Overall, diverse key genes regulate seed germination by mediating ABA and GA biosynthesis and/or signal transduction pathways (**Table 1**).

**Table 1:** General descriptions of recent reported factors roles in plant development and abiotic stress responses in regard to ABA and GA crosstalk network.

Developmental stages or abiotic stress	Genes or general description	Species	References
Seed dormancy and germination	ABI4 is the central factor between ABA and GA crosstalk.	*Arabidopsis*	Shu et al., 2013, 2016b,c, 2018b; Li et al., 2014; Wang et al., 2014; Huang et al., 2017
	SbABI4 is involved in seed dormancy.	*Sorghum bicolor*	Cantoro et al., 2013
	GIM2 is involved in ABA and GA balance.	*Arabidopsis*	Xiong et al., 2017
	Auxin and NaCl delay soybean seed germination through decreasing the ratio of GA/ABA	*Glycine max*	Shu et al., 2017; Shuai et al., 2017
	NaCl inhibits seed germination in *Suaeda salsa* seeds through ABA and GA pathways.	*Suaeda salsa*	Li et al., 2015
	Exogenous H_2_ alleviated the Al toxicity through increasing the GA/ABA ratio.	*Oryza sativa*	Xu et al., 2017
	SA and H_2_O_2_ increase GA/ABA ratio and accelerate maize seed germination under chilling conditions.	*Zea mays*	Li Z. et al., 2017
	Karrikins mediate soybean seed germination by regulating GA and ABA balance.	*Soybean*	Meng et al., 2016a,b
	NF-YC-RGL2-ABI5 cascade integrates GA and ABA signaling pathways.	*Arabidopsis*	Liu X. et al., 2016
	FOA2, FUSCA3, DAG1.	*Arabidopsis*	Boccaccini et al., 2014a,b, 2016; Chiu et al., 2016a,b; He et al., 2016
Root development	Low concentration of ABA enhances quiescence of the quiescent center and suppresses stem cell differentiation.	*Arabidopsis*	Zhang et al., 2010
	ABA inhibits root growth by enhancing ethylene biosynthesis.	*Arabidopsis*	Luo et al., 2014
	HDT1/2 mediate the switch from cell division to expansion in the root tip through repressing the transcription of *GA2ox2.*	*Arabidopsis*	Li H. et al., 2017
	ABA and GA function together to mediated periclinal asymmetric cell divisions.	*Arabidopsis*	Cui and Benfey, 2009.
	GAZ is involved in ABA and GA homeostasis during root ground tissue formation.	*Arabidopsis*	Choi and Lim, 2016; Lee et al., 2016
	SEUSS is involved in the regulation of the expression of *SHR*, *SCR*, and *SCL3*, and transcription of *SEUSS* was repressed by GA.	*Arabidopsis*	Gong et al., 2016
Flowering time control	ABI4 and ABI5 negatively regulate plant flowering time.	*Arabidopsis*	Wang et al., 2013; Shu et al., 2016a
	Positive effect of ABA on flowering time.	*Arabidopsis*	Riboni et al., 2013, 2016
	Controversial effect of ABA on flowering.	*Arabidopsis, Oryza sativa*	Shu et al., 2018a
Shade response	The effect of GA on shade adaptation in perennial ryegrass.	*Lolium perenne*	Li W. et al., 2017
	GA positively regulates plant shade avoidance.	*Arabidopsis*	Liu H. et al., 2016
	BBX24 promotes plant shade avoidance response through attenuating DELLA activity.	*Arabidopsis*	Crocco et al., 2015
	Shade stress up-regulates ABA level in tomato and sunflower.	*Solanum lycopersicum Helianthus annuus*	Kurepin et al., 2007; Cagnola et al., 2012
	Shade promotes the transcription of ABA biosynthesis genes *NCED3* and *NCED5*, and of ABA signaling genes, *ABF3.*	*Arabidopsis*	Kohnen et al., 2016; Sellaro et al., 2017

## ABA and GA in the Regulation of Root Development

The root system is of great significance in both plant stress response and nutrient absorption. Numerous studies have demonstrated that auxins play important roles in the regulation of root growth, especially in the maintenance of the root stem cell niche (Liu et al., 2017; Du and Scheres, 2018). However, ABA and GA are also involved in the control of root development, although the detailed molecular mechanisms involved require further investigation.

A previous study had revealed that low concentrations of ABA enhanced quiescence of the quiescent center and suppressed stem cell differentiation in the primary root meristem niche of *Arabidopsis* (Zhang et al., 2010). Applications of high concentrations of exogenous ABA or the abiotic stress-induced accumulation of ABA inhibited *Arabidopsis* primary root growth, but the molecular mechanisms involved are not fully understood. An earlier study showed that ABA promoted the transcription of *ICK1/KRP1,* which encode a negative regulator of the cell cycle, so that ABA delays cell expansion and proliferation (Wang et al., 1998). Recently, the Gong lab showed that ABA inhibited root growth by enhancing ethylene biosynthesis (Luo et al., 2014). The ethylene biosynthesis inhibitor L-alpha-(2-aminoethoxyvinyl)-glycine reduced ABA-mediated inhibition of root growth. Further biochemical analysis revealed that CPK4 and CPK11, two ABA-activated calcium-dependent protein kinases, phosphorylate the C-terminus of ACS6, increasing the stability of this protein, and promote ethylene biosynthesis (Luo et al., 2014). The identification of this ABA-ethylene cascade represents a recent breakthrough in the regulation of root development mediated by ABA.

Previous studies had demonstrated that GA exhibited a positive effect on root growth in *Arabidopsis* (Ubeda-Tomas et al., 2008, 2009). A recent study revealed that HDT1/2 (histone deacetylases) mediated the switch from cell division to expansion in the root tip through repressing the transcription of *GA2ox2*, a GA-inactivator gene (Li H. et al., 2017). Further genetic analysis showed that upregulation of *GA2ox2* in *hdt1* and *hdt2* background caused a decrease in GA concentration, which then resulted in an earlier switch from cell division to cell expansion of the transit-amplifying cells developing from the root stem cells (Li H. et al., 2017). Precise control of the timing and extent of asymmetric cell divisions is crucial for correct patterning. Previous studies had demonstrated that ABA and GA function together to mediate periclinal asymmetric cell divisions of the endodermis during ground tissue formation (Cui and Benfey, 2009). Recent studies revealed that the *GAZ* (*GA- AND ABA-RESPONSIVE ZINC FINGER*) gene is involved in the regulatory pathways, while transcription of *GAZ* is regulated by GA and ABA (Lee et al., 2016). Transgenic *GAZ*-overexpressed plants were sensitive to both ABA and GA during the middle cortex formation stage, whereas *RNAi-GAZ* lines displayed the opposite phenotype. Further transcriptional analysis showed that GAZ is also involved in ABA and GA homeostasis during root ground tissue formation (Choi and Lim, 2016; Lee et al., 2016).

In addition to GAZ, another key factor, SEUSS, is also involved in middle cortex formation. The *seu* mutants exhibited clearly reduced expression of *SHR* (*SHORT-ROOT*), *SCR* (*SCARECROW*), and *SCL3* (*SCARECROW-LIKE3*), suggesting that SEUSS positively regulated the transcription of these genes (Gong et al., 2016). In addition, *SEUSS* transcription was repressed by GA and enhanced by the GA biosynthesis inhibitor, paclobutrazol, indicating that SUESS regulated middle cortex formation via the GA pathway (Gong et al., 2016) (**Table 1**). However, the antagonism between ABA and GA in the regulation of root initiation needs further exploration and dissection.

## Flowering Time Control: the Other Battlefield Between ABA and GA

During the plant life cycle, the appropriate flowering time is a crucial and important checkpoint for growth and survival, especially under diverse environmental stress conditions. The positive effect of GA on plant floral transition has been extensively and intensively explored and documented (Ding et al., 2013; Hyun et al., 2016; Zhu et al., 2016; Brambilla et al., 2017; Conti, 2017; Gong et al., 2017; Sawettalake et al., 2017).

ABA is also involved in the regulation of flowering time (Wang et al., 2013; Shu et al., 2016a). However, the contribution of ABA to the control of flowering time is still controversial, as both positive and negative effects have been documented (Riboni et al., 2013, 2016; Wang et al., 2013; Shu et al., 2016a). With regard to the effect of ABA on floral transition, we have recently reviewed the literature and proposed a working model (Shu et al., 2018a). The positive and negative effects of ABA on floral transition may be associated with environmental cues, such as drought, salt and other abiotic stresses (Riboni et al., 2016; Shu et al., 2018a) (**Table 1**). However, the detailed mechanisms through which ABA and GA antagonistically mediate flowering need further investigation.

## Shade Response: the New Battlefield Between ABA and GA?

Numerous investigations have demonstrated that ABA is involved in diverse abiotic/biotic stress response cascades, including drought, salt, low temperature and pathogens, with many research articles and reviews covering the effects of ABA on stress responses (Edel and Kudla, 2016; Skubacz et al., 2016; Lievens et al., 2017; Saradadevi et al., 2017). Although GA is primarily regarded as a hormone contributing to the control of plant growth and development, recent data showed that GA also plays roles in plant adaptation to stresses, such as dehydration stress (Qin et al., 2011; Plaza-Wuthrich et al., 2016; Urano et al., 2017). A negative regulator of GA signaling, SPINDLY, plays a negative role in drought stress tolerance by integrating GA and cytokinin crosstalk (Qin et al., 2011). Under early osmotic stress, differential levels of GA biosynthesis gene expression, DELLA-regulated transcription and RGA protein accumulation were reported in proliferating cells (Skirycz et al., 2011; Claeys et al., 2012). GA is also involved in plant shade and flooding stress responses (Bailey-Serres and Voesenek, 2010; Colebrook et al., 2014; Li W. et al., 2017). In this review, we focus mainly on the effects of ABA and GA on shade response.

Shade stress is expressed as reductions in both photosynthetically active radiation and the ratio between red and far-red (R/FR) light, resulting from sunlight passing through the leaves of neighboring plants under dense planting systems (Yang and Li, 2017; Yang et al., 2018). Auxin biosynthesis and signal transduction plays a key role with regard to plant shade response, and the phytochromes PHYA, PHYB, and the phytochrome-interacting factors (PIFs) are involved in these cascades (Casal, 2012, 2013; Yang et al., 2018). A recent study detected an effect of GA on shade adaptation in perennial ryegrass (*Lolium perenne*) (Li W. et al., 2017). The ryegrass *shadow-1* mutant exhibited the dwarf and shade-insensitive phenotype, while transcriptome analysis revealed that the transcription of GA biosynthesis and response genes was down-regulated in *shadow-1* plants, compared to the wild type (Li W. et al., 2017). This study highlighted the important roles of GA biosynthesis under shade conditions. In line with this, phenotypic analysis of GA-related mutants also suggested that GA positively regulated plant shade avoidance in *Arabidopsis* (Liu H. et al., 2016). Another study demonstrated that the transcriptional regulator BBX24 promoted the plant shade avoidance response through attenuating the activity of DELLA proteins, negative regulators of the GA signaling pathway (Crocco et al., 2015). The shade-response defect in *bbx24* mutants was fully restored by exogenous GA application, a treatment which promotes DELLA degradation (Crocco et al., 2015). These investigations highlighted the important functions which GA play in the plant shade response (**Table 1**).

Although ABA is involved in diverse abiotic stress response pathways, the detailed roles of ABA in shade avoidance have not been elucidated to date. Earlier studies had shown that shade stress up-regulated ABA concentration in tomato and sunflower (Kurepin et al., 2007; Cagnola et al., 2012). Recent studies revealed that shade stress promoted the transcription of several ABA biosynthesis genes, including *NCED3* and *NCED5*, and of the ABA signaling gene, *abscisic acid-responsive element-binding factor 3* (*ABF3*) (Kohnen et al., 2016; Sellaro et al., 2017). However, a better understanding of the function of ABA in the regulation of shade response is needed.

## Concluding Remarks

The antagonism between ABA and GA in the control of diverse aspects of plant development and abiotic stress response is an attractive target in the research field of plant molecular biology. Significant progress has been made in the model plant *Arabidopsis* to understanding the underlying mechanisms. However, several key questions remain to be answered.

Firstly, it is proposed that several key factors regulate the balance between ABA and GA, and subsequently achieve precise mediation of plant development and stress responses. Several transcription factors, including ABI4 and OsAP2-39, belong to this large family, which directly or indirectly controls the transcription pattern of ABA and GA biosynthesis genes (Yaish et al., 2010; Shu et al., 2013, 2016b). Identification and dissection of the modes-of-action of other, currently unknown transcription factors, which mediate ABA and GA antagonism, would be a major step forward in GA/ABA antagonism research, while identification of the target genes of these transcription factors in ABA and GA biosynthesis and/or signaling pathways would also be a most worthwhile project.

Secondly, in addition to transcriptional control, regulation at the post-transcription level also needs an increased focus, whereby the currently unknown transcription factors regulate ABA and GA antagonism through mediating the activity of some of the enzymes involved in ABA- and GA- related biosynthesis and signaling pathways. For instance, different types of protein modification, including protein ubiquitination, acetylation, methylation and phosphorylation, affect ABA/GA biosynthesis and signal transduction, contributing to the control of plant development and stress responses.

Thirdly, although GA has been shown to positively regulate plant shade response (Liu H. et al., 2016; Li W. et al., 2017), the detailed mechanisms involved, especially the relationship between GA and auxin, need further investigation. In addition, the roles of ABA in plant response to shade conditions are also not fully understood. Overall, these remaining scientific questions with regard to ABA and GA antagonism are worthy of further exploration.

## Author Contributions

KS conceived and designed this paper and wrote the manuscript. KS, WZ, FC, and XL sorted and discussed those published papers. KS and WY analyzed the database.

## Conflict of Interest Statement

The authors declare that the research was conducted in the absence of any commercial or financial relationships that could be construed as a potential conflict of interest.
